# Alternative reproductive tactics and evolutionary rescue

**DOI:** 10.1093/evlett/qrae010

**Published:** 2024-03-16

**Authors:** Robert J Knell, Jonathan M Parrett

**Affiliations:** School of Natural Sciences, University of Hull, Hull, United Kingdom; Evolutionary Biology Group, Faculty of Biology, Adam Mickiewicz University, Poznań, Poland

**Keywords:** adaptation, changing environments, “sneak” matings, polyphenism, sexual selection, mating systems

## Abstract

Almost all life on earth is facing environmental change, and understanding how populations will respond to these changes is of urgent importance. One factor that is known to affect the speed by which a population can evolve when faced with changes in the environment is strong sexual selection. This increases the adaptive capacity of a population by increasing reproductive skew toward well-adapted (usually) males who will, on average, be best able to compete for matings. This effect could potentially be disrupted when males pursue alternative reproductive tactics (ARTs), whereby males within a species exhibit qualitatively different behaviors in their pursuit of matings. ARTs are diverse, but one common class is those expressed through condition-dependent polyphenism such that high-quality, well-adapted males compete aggressively for mates and low-quality, poorly adapted males attempt to acquire matings via other, nonaggressive behaviors. Here, using an individual-based modeling approach, we consider the possible impacts of ARTs on adaptation and evolutionary rescue. When the ART is simultaneous, meaning that low-quality males not only engage in contests but also pursue other tactics, adaptive capacity is reduced and evolutionary rescue, where a population avoids extinction by adapting to a changing environment, becomes less likely. This is because the use of the ART allows low-quality males to contribute more maladaptive genes to the population than would happen otherwise. When the ART is fixed, however, such that low-quality males will only use the alternative tactic and do not engage in contests, we find the opposite: adaptation happens more quickly and evolutionary rescue when the environment changes is more likely. This surprising effect is caused by an increase in the mating success of the highest quality males who face many fewer competitors in this scenario—counterintuitively, the presence of males pursuing the ART increases reproductive skew toward those males in the best condition.

## Introduction

Organisms usually exist in habitats that they are reasonably well adapted to. When the environment changes such that those organisms suffer substantial reductions in fitness, there are several possible outcomes: The population in question might become extinct, or the organisms might migrate to or otherwise colonize new environments that have become more suitable to them. A third possibility is “evolutionary rescue”: the phenomenon whereby the affected population evolves rapidly enough to become adapted to the new environment and thus is able to persist despite the environmental changes (Bell, 2017). Evolutionary rescue will only occur when the rate of adaptation is sufficient to offset the fitness reductions arising from environmental change. Understanding the factors that determine how fast adaptation can occur is therefore necessary for predicting responses to changing environments. Given that the environment is changing rapidly in almost every habitat on the planet, and many populations have a limited ability to migrate due to geographical barriers or habitat fragmentation, this is now of considerable importance.

Over recent decades, it has become clear that mating systems can be important determinants of adaptive capacity for animal populations—there is now firm evidence from laboratory studies that strong sexual selection can enhance adaptation and therefore the probability of evolutionary rescue (Cally et al., 2019; Godwin et al., 2020; Jarzebowska & Radwan, 2010; Parrett & Knell, 2018; see Parrett et al., 2019 for a field example). These positive effects of strong sexual selection are a consequence of sexual signals expressed by (usually) males being condition dependent (Dougherty, 2021; Rowe & Houle, 1996; Tomkins et al., 2004), meaning that they respond disproportionately to the overall health and well-being of the bearer. If females base their mating decisions on these signals, or if male–male contests are determined on the basis of their expression, then males that are well adapted to the new environment, and therefore in better condition, will gain a high proportion of matings and father a higher proportion of the offspring in the next generation than would otherwise occur, leading to more rapid adaptation and potentially a greater probability of evolutionary rescue (Agrawal & Whitlock, 2012; Lorch et al., 2003; Martínez-Ruiz & Knell, 2017; Whitlock, 2000).

Mating systems are, of course, greatly diverse and strong sexual selection with either female-choice or intrasexual contests, leading to high reproductive skew in favor of a few high-quality males is only one of many possible variants, each of which could have differing effects on the rate of adaptation and the potential for evolutionary rescue. Here we consider the potential role of one widespread variant, whereby males adopt alternative reproductive tactics (ARTs); either competing with rivals for access to mates (variously called “major,” “fighter,” “bourgeois,” or “guarder” males) or attempting to acquire matings by means other than direct competition (“minor,” “scrambler,” “parasitic,” or “sneak” males) (Engqvist & Taborsky, 2016; Gross, 1996; Neff & Svensson, 2013; Oliveira et al., 2008). ARTs are diverse and distributed across the animal kingdom. Some, such as those found in the ruff, *Philomachus pugnax* (Lamichhaney et al., 2016; Lank et al., 1995), and the side-blotched lizard, *Uta stansburiana* (Sinervo & Lively, 1996), are largely determined by genetic polymorphisms associated with specific mating phenotypes, with the polymorphism often being maintained by frequency-dependent selection. Others, however, are polyphenisms, whereby any single genotype can develop into multiple phenotypes. In the dung beetle *Onthophagus taurus*, e.g., males either develop into horned “major” males or effectively hornless “minor” males depending on food availability when larvae (Hunt & Simmons, 1997; Moczek & Emlen, 1999). The horned major males guard females in tunnels and will fight other major males who intrude, but minor males do not fight and instead attempt to acquire matings using nonaggressive tactics including mating with females when they leave the burrow to collect dung or passing a guarding male without fighting to access the female beetle (Moczek & Emlen, 2000).

In general, the polyphenic expression of morphs, which engage in alternative tactics, is thought to be an example of conditional expression, whereby individuals that are small, weak, or otherwise of low status adopt alternative, nonaggressive mating tactics because these tactics lead to higher fitness for them. Large, strong, or otherwise high-status males, on the other hand, gain the greatest fitness benefits from competing aggressively (Gross, 1996; Hunt & Simmons, 2001; Tomkins & Hazel, 2007). Since the generally accepted explanation for the mechanism by which sexual selection enhances adaptation is based on the idea that males in good condition (high status) acquire the majority of matings, it might be expected that this effect should be disrupted by the expression of ARTs since these increase the mating success of males in poor condition, weakening selection against deleterious alleles or for beneficial ones.

This potential effect of ARTs on adaptation is, of course, dependent on ART expression having a heritable component such that those individuals expressing the ART are indeed poorly adapted or carrying a high mutational load when compared with those individuals pursuing the more competitive reproductive tactic. The genetics of ART allocation in most condition-dependent polyphenic systems are not well studied apart from the bulb mite, *Rhizoglyphus robini* (Parrett et al., 2023, 2022; Radwan, 1995; see also Buzatto et al., 2012 for a further quantitative genetic example from a congeneric). In this species, morph allocation is a complex product of environmental (Smallegange, 2011) and genetic factors, with considerable additive genetic variance, which is likely in part linked to mutational load (Parrett et al., 2023).

Whether this is the case in other, similar ART systems is currently unknown. Studies of polyphenic beetles have yielded mixed results, with some finding of no significant heritability in morph allocation (*O. taurus*: Moczek & Emlen, 1999; *Trypoxylus dichotomus*: Karino et al., 2004). Both of these studies used quite small sample sizes, however (*n* = 17 and *n* = 18, respectively). By contrast, a selection study on *Onthophagus acuminatus* indicates that morph allocation is heritable (Emlen, 1996), and field observations of rapid evolution in morph allocation in *O. taurus* (Moczek et al., 2002) also indicate an important genetic component to morph allocation.

More widely, a heritable component to morph allocation linked to condition seems a reasonable assumption for most polyphenic systems. Condition dependence in sexually selected trait expression, as proposed by Rowe and Houle (1996), is widely thought to reflect mutational load and/or maladaptation (e.g., Dugand et al., 2019), and it seems unlikely that this will also not be captured in the discrete and nonlinear expression of sexually selected traits in species with polyphenic ARTs.

Here, we develop and analyze an individual-based model (IBM) of populations evolving in a changing environment, with males able to adopt either a competitive or a noncompetitive strategy on the basis of their condition—thus, we consider only the status-dependent case of ARTs with polyphenic males and not the very different frequency-dependent variant, nor do we consider sequential ARTs whereby tactics alter depending on age. IBMs are a useful approach to explore complex evolutionary processes, which interact with demography because they allow individual variation to be explicitly included (DeAngelis & Mooij, 2005).

We distinguish between “simultaneous” and “fixed” ARTs (Taborsky et al., 2008) where simultaneous ARTs can be expressed at the same time as the “normal” tactic by the same individual, as in three-spine sticklebacks where nest-holding males will also engage in sneak tactics (Rico et al., 1992), and fixed ARTs are those where one individual is only able to express one tactic, such as *O. taurus* and *R. robini* examples given above. Population persistence under directional environmental change and the speed of return to a near-optimal level of adaptation after a step change in the environment are both modeled.

## Methods

The model is coded in R (R Development Core Team, 2022). It simulates the dynamics and evolution of a spatially homogeneous, age-structured population of animals. The model proceeds as a series of discrete time steps of arbitrary length. All simulations described here were run for 500 time steps or until the population became extinct, whichever was shorter. Multiple simulations were run in parallel using the doparallel (Microsoft Corporation & Weston, 2022a) and foreach (Microsoft Corporation & Weston, 2022b) packages running in R version 4.3.0 on a 2021 MacBook Pro with a 10-core M1 Pro CPU and 32GB RAM.

The environment is described by a single continuous variable *environment*, which is best thought of as representing an environmental variable such as temperature or pH. The value for *environment* is initially set at 1, and two different treatments were used: directional change and step change. For directional change, the environment was held constant aside from a small amount of random variation for the first 25% of the time steps. Following this and for the rest of the simulation, the environment changes in a directional way, with a value drawn from a normal distribution with *μ* equal to a parameter *directional rate* and *σ* = 0.005 added to the value at each time step. For the step-change treatment, the environment was allowed to remain constant aside from a small amount of random variation, but at time point 100, the value of *environment* had 0.33 added. This change is not sufficient to make the population become extinct, but populations exposed to such an environmental change will be some distance from being optimally adapted once the change has happened.

Populations are started with 200 individuals, which are allocated the following characteristics: *sex* (male or female, allocated at random); *age* (a value from 1 to 10, drawn at random from a uniform distribution); *environmental genotype*, which determines how well that individual is adapted to the environment (drawn for each individual from a normal distribution with *σ* = *0.25* and *μ* = the starting value for the “environment” variable); and *resource*, drawn at random from a uniform distribution with minimum 0 and maximum 1, which describes how each individual is able to acquire resources independently of the specific environmental effect being modeled. Including *resource* in the model here adds an element of environmental variance to the expression of the sexual trait, which in real systems will likely be determined by both environmental and genetic variance (Singh & Agrawal, 2022).

Each individual’s condition is then calculated as:


$$\begin{array}{l} Condition\;=\;resource-\left|environmental\;genotype-environment\right| \end{array}$$

Individuals with *condition* < 0 die, so *condition* is a value between 1 and 0 for all living individuals.

The variable *threshold* determines whether a male with a given value for *condition* will develop into a major male (aggressive strategy) or a minor male (nonaggressive strategy). A fixed threshold for morph determination is consistent with the status-dependent model (Gross, 1996) and is consistent with what we know about morph allocation in animals such as the European earwig *Forficula auricularia* (Tomkins, 1999) and dung beetles from the genus *Onthophagus* (Emlen, 1997; Hunt & Simmons, 1997; Moczek & Emlen, 1999). As discussed in the Introduction section, there is uncertainty regarding the extent by which morph allocation reflects the genetic makeup of the males, and including the *resource* variable in the calculation means that there is a strong element of morph determination that is not a product of the individual’s genotype. Simulations indicate that when a population is well adapted (i.e., the population mean value for *environmental genotype* is equal to the *environment* variable), then *resource* explains about 10 times as much of the variation in morph as does the mismatch between genotype and environment. When the population is not well adapted (in this case with a difference of 0.4 between *environment* and *environmental genotype*), then *resource* explains about twice as much of the variance in morph than is explained by the mismatch between genotype and environment. We explore the impact of reducing the genetic contribution to morph allocation further in Supplementary Material.

In our model, the strategy a male follows is determined at *age* = 1 and then fixed for the lifetime of that individual, so this aspect of the model is representative of ARTs in examples such as bulb mites, dung beetles, and salmon where the alternative tactic is determined before sexual maturity. If *threshold* = 0, all males follow the aggressive strategy. Major males over the age of maturity (set at 2) express a display trait (*display*), which is equal to their *condition*. Minor males do not express such a trait. Two versions of the ART were used: one where the strategy is fixed (sensu Taborsky et al., 2008), and minor males do not attempt to compete for matings, and one where the strategy is simultaneous and minor males will compete for matings but will also try to acquire matings by other means.

Every time step, the *age* for each individual is incremented by 1. Following this, the probability of dying is calculated for each individual as:


$$\begin{array}{l} p\;(death)=\frac{N_{t}}{K}\;+\;cost.display+0.0154.age^{2}+0.169.age+0.46 \end{array}$$

where *N*_*t*_ = *population size*, *K* determines the maximum population size, *cost* is the mortality cost of engaging in contests or energetically expensive displays, plus the costs of bearing the display trait. These are known to be substantial in many species (Clutton-Brock et al., 1997; Coulson et al., 2001; Liker & Székely, 2005; Loveridge et al., 2007; Promislow, 1997). Females and minor males do not express the display trait and do not pay this additional mortality cost: see Supplementary Material for a discussion of the effect of relaxing this such that major males pay a reduced or no cost. The quadratic component of the equation gives higher mortality for young and old individuals, with the coefficients having arbitrary values that gave an appropriate scale and shape of curve.

For mate allocation, males are divided into groups with size determined by a parameter *g*. If the ART is fixed, then only major males are allocated to these groups, if simultaneous then all males above the age of maturity are allocated to “mating groups” within each of which they compete for access to females for mating. The males in each group are ranked on the basis of the value of their display trait, and each female of reproductive age is allocated to one of the groups of males.

Each female will mate with one male from the group she is allocated to. The probability of mating is calculated for each male in a group on the basis of his ranking and the value of a parameter *β* as follows:


$$\begin{array}{l} p(mate)\;=\;\frac{1}{rank^{\beta }}/\sum_{x=1}^{g}\frac{1}{x^{\beta }}\; \end{array}$$

*β* controls the relationship between display trait expression and mating—this can be thought of as either the strength of female preference or the strength of the advantage in contests between males given by the display trait, with higher values of *β* meaning that males with large display traits have a greater advantage.

Condition is likely to have some effect on mating success in minor males, but this is likely to be a much weaker effect than in majors (e.g., Hunt & Simmons, 2001), and for simplicity, we assume that mating success in minor males is not associated with condition. Following the allocation of mates via contests between males, each female can also mate with a randomly selected minor male with a probability equal to the proportion of minor males multiplied by a parameter *ART_success*, which allows the success of the minor strategists to be adjusted. Each female then produces a number of offspring equal to the maximum offspring multiplied by her condition, rounded to the nearest integer. For females who mate with both a major and a minor male, paternity for each offspring is allocated at random with equal probability for both males.

The *environmental genotype* is modeled as a quantitative, polygenic trait controlled by many alleles, so each offspring has an *environmental genotype* equal to the mean of its parents’ *environmental genotype* value plus a value drawn from a normal distribution with *mean* = *0* and *σ* equal to a parameter called *mutation.*

For simulations run with directional change, the response variable used was whether the population became extinct over the course of the simulation. When the environmental change was a step change, the response variable used was the number of time units after the change before the upper quartile of the distribution of values of *environmental genotype* first became equal to or greater than the *environment* variable. This gives an indication of how long it takes the population to adapt to the new value of *environment*.

Supplementary Material contains output from simulations exploring the effects of reducing the additional mortality costs of expressing and signal, and of reducing the genetic contribution to morph allocation. It also has details of a further set of analyses exploring the effect of variables including the ART type, mating group size, *β*, and *ART_success* in determining the Critical Rate of Environmental Change (Chevin et al., 2010) for these simulated populations.

## Results

### Directional change

**Figure 1.**
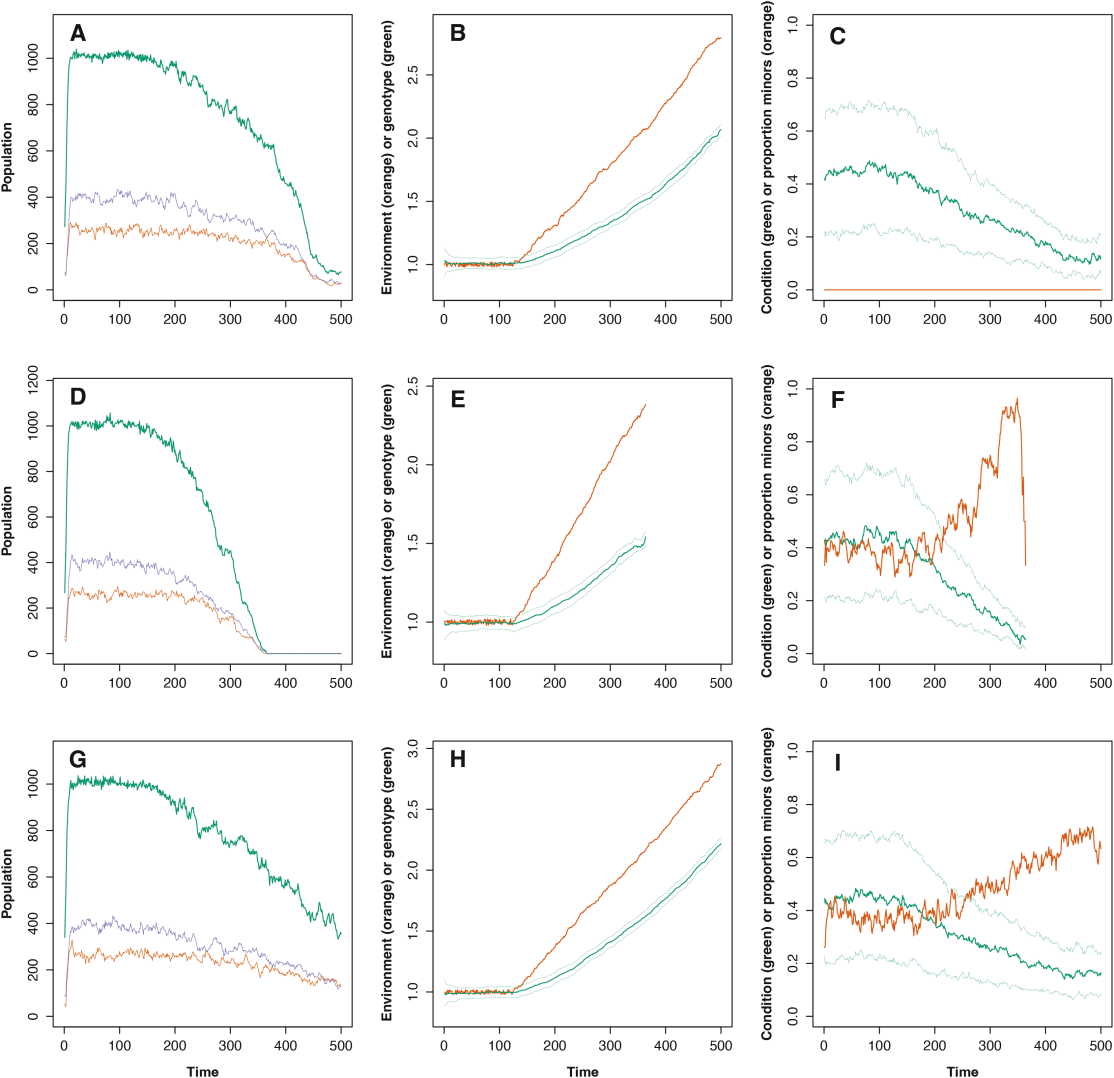
Representative model output. (A–C) All males follow the “major strategy”—in other words, there are no alternative tactics. (D–F) Male alternative tactics are simultaneous. (G–I) Male alternative tactics are fixed. (A, D, G) Total population (green, upper line in all cases) plus the populations of mature females (purple, middle line) and males (orange, lower line). (B, E, H) The value of the *environment* variable at each time step (orange, upper line) plus the median and quartiles of the *environmental genotype* variable (green, lower line). (C, F, I) The median and quartiles of *condition* (green, line with associated paler lines for quartiles) plus the proportion of males adopting the minor strategy (orange, lower ine in C, F & I the line which is higher towards the right hand side of the plot). Parameter values for the simulations are *K* = 1,000, *threshold* = 0.25, *max_offspring* = 6, *group_size* = 6, *ART_success* = 0.5, and *β* = 2. For the two cases where alternative tactics were available, *threshold* = 0.25; for the case when no alternative tactics were followed, *threshold* = 0*.*

Figure 1 shows the typical output from the model when the environment changes in a directional fashion for simulations with no alternative tactics expressed (Figure 1A–C), simultaneous alternative tactics (Figure 1D–F), and fixed alternative tactics (Figure 1G–I). While the environment remains roughly constant, the population fluctuates around the carrying capacity. There is some skew in the adult sex ratio as a consequence of the extra mortality cost experienced by major males, and when alternative tactics are expressed, the proportion of minor males in the population is roughly 40% (Figure 1F and I). At *time* = 125, the environment starts to undergo directional change. In each case, the population evolves in response, as can be seen by the increasing value of *environmental genotype* (the variable that determines the optimal value for the environment for each individual) in Figure 1B, E, and H. In all cases, the rate at which the population can adapt is slower than the rate of change of the environment and the median condition of the population declines (Figure 1C, F, and I), leading to both an increased death rate and lower reproduction by females. When alternative tactics are expressed, the proportion of minor males increases as a consequence of the generally lower condition of the population. In the case of simultaneous reproductive tactics, extinction occurs after roughly 350 time steps, but for the no alternative tactics and fixed alternative tactics examples the population, while much reduced, persists until the end of the simulation. The gap between the median *environmental genotype* value and the median *environmental* value is an indication of the difference between population adaptation and the environment, and in Figure 1B and H, this gap can be seen to be less compared to Figure 1E, indicating that the population with simultaneous alternative tactics is not able to adapt as fast as the other two populations

**Figure 2.**
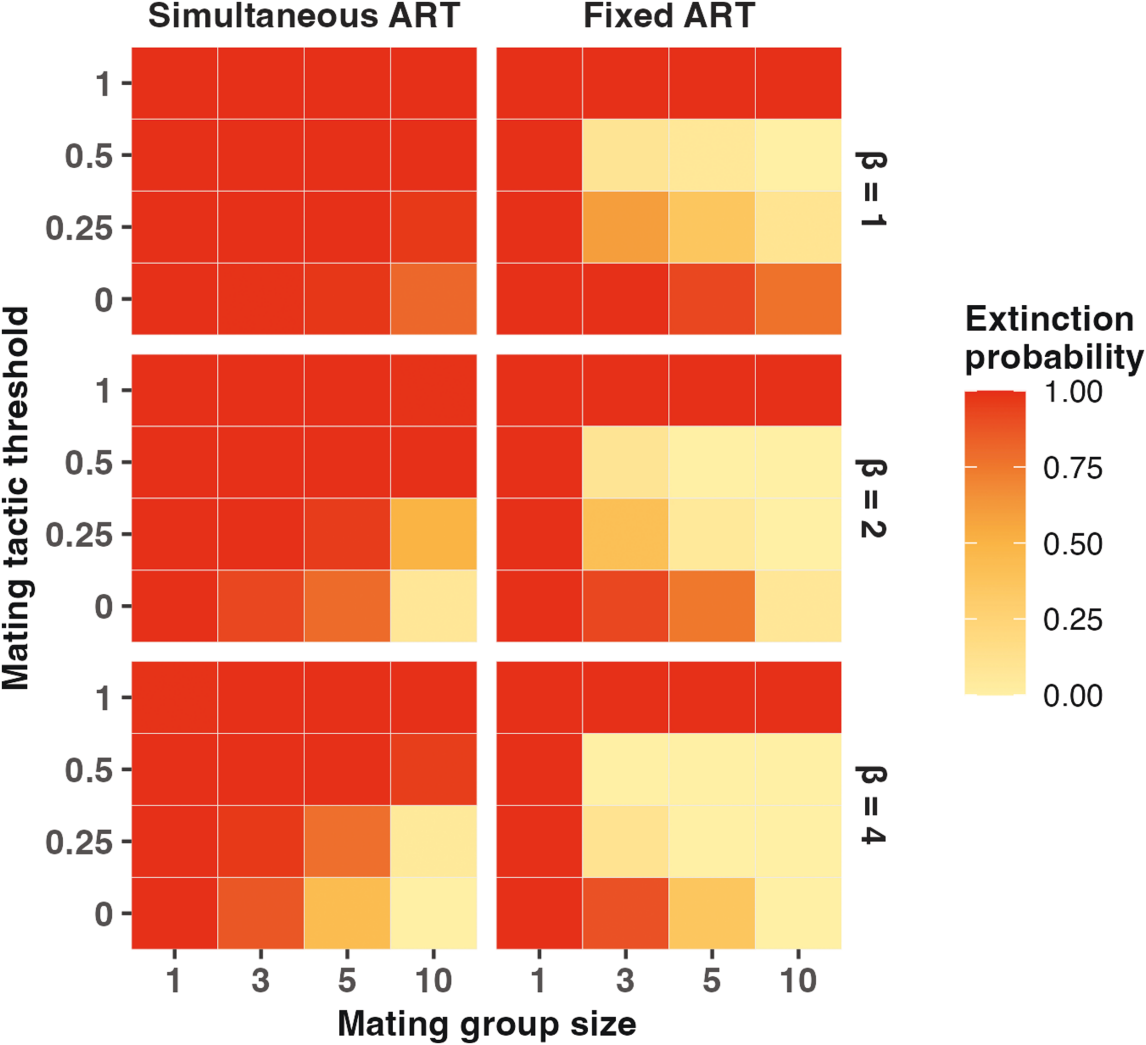
Heatmap showing the probability of extinction under directional environmental change when alternative tactics are simultaneous (left-hand column) or fixed (right-hand column), calculated from 100 simulations running for 500 time steps for each combination of parameter values. The rate of directional change was set to 0.005 for all simulations. Other parameter values: *max_offspring* = 6, *ART_success* = 0.5, *K* = 500.

Figure 2 shows the effect of alternative tactics on extinction probability for both fixed and simultaneous ARTs. In these simulations, the rate of directional change was set to a single value, which was sufficient to cause extinction in all simulations when mating was random—in this model, random mating occurs when either the mating group size or the threshold condition value (the value for *condition* which determines whether males follow the ART or not) for pursuing the alternative mating tactic threshold is 1. If there is relatively strong sexual selection and no males pursuing alternative strategies (mating tactic threshold = 0, mating group size > 1 and *β* > 1), then evolutionary rescue can occur, especially when the population is large and sexual selection is strong (mating group size is 10 and *β* = 2 or 4), in which case all populations are able to persist over the course of the simulation. The inclusion of some males pursuing simultaneous alternative tactics (left-hand column, mating tactic threshold = 0.25 or 0.5), however, negates this effect of sexual selection, and the probability of evolutionary rescue of these populations is reduced substantially.

By contrast with the situation for simultaneous alternative tactics, when the alternative tactics are fixed and minor males do not compete in groups for access to females, the presence of males pursuing alternative tactics increases the probability of evolutionary rescue. The right-hand column of Figure 2 shows the proportion of simulations where the population became extinct for a range of parameter values with fixed alternative tactics. When some lower condition males pursue the alternative tactic and do not compete with the other males, evolutionary rescue is more likely, occurring at lower values of *β* and mating group size than when males do not pursue the alternative tactics, and in smaller populations. In this case, the median value of *environmental genotype* can keep pace with the increasing value of *environment* and the population persists, albeit at a size far below the carrying capacity and with most individuals in poor condition.

### Environmental step change

**Figure 3.**
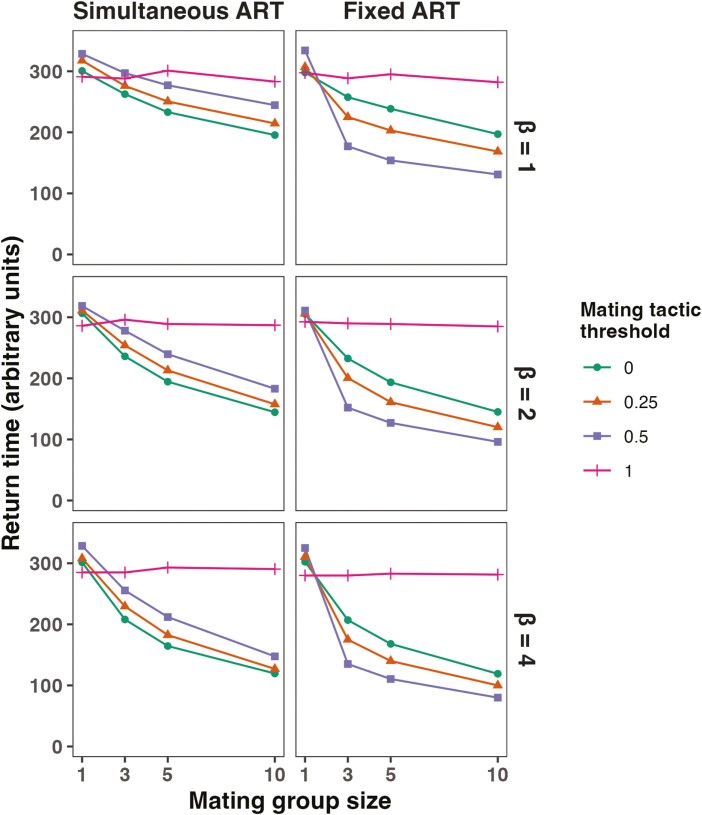
Median return times for populations following an environmental step change. Return times are the number of time units before the upper quartile of the distribution of *environmental genotype* is equal to the value of *environment.* When the mating tactic threshold = 1, all males follow the ART, meaning that there is random mating in the simulation and males do not express sexual signal traits. Each value is the median from 100 simulations, and the carrying capacity was set at 500 for all simulations.

Median return times for simulations that underwent an environmental step change are shown in Figure 3, with the return time here being the number of time steps after the step change before the upper quartile of the distribution of values for the *environmental genotype* became equal to or greater than the value for *environment*. The strength of sexual selection increases with both the mating group size and the parameter determining the strength of female choice or the advantage of higher quality males in contests within a group (*β*) and the return time declines as both of these increase, indicating faster adaptation. When the alternative tactics are simultaneous, the presence of males following alternative tactics leads to slower return times and when males are more likely to follow the ART (*threshold* = 0.5 rather than 0.25), there is a greater increase in return time and therefore slower adaptation. When the ART is fixed, however, the inverse is found and the presence of males following the ART leads to shorter return times and therefore faster adaptation. Thus, consistent with the results from the directional change case above, simultaneous ARTs reduce adaptation, whereas fixed ARTs increase it.

When the mating tactic threshold = 1, then all males follow the ART, and so the return time is unaffected by the strength of sexual selection. Interestingly, when sexual selection is very weak or nonexistent (group size = 1), then the return times for the cases when all males follow the ART are actually lower than the alternatives. This is most probably because in this model males following the ART do not express the sexual signal and so do not pay a cost for doing so, whereas even when the group size = 1, males not following the ART will pay the cost for expressing the signal. Well-adapted, high-quality males will therefore have a somewhat longer life in the simulations where all males follow the ART and will consequently produce more offspring.

### Reproductive skew with simultaneous and fixed ARTs

Why do simulations with fixed ARTs show faster adaptation and an enhanced probability of evolutionary rescue? In this case, the low-condition males are excluded from the mating groups where males compete for matings. If all males are allocated to these groups, as when there are no males pursuing alternative tactics or when the alternative tactics are simultaneous, then purely by chance some groups will have no high-condition males, giving the low-condition males in those groups an opportunity to mate. If low-condition males do not compete, then all the groups of competing males will consist of high-condition males, enhancing the probability of evolutionary rescue because under these conditions all the females will mate with a relatively high-condition male, who most likely will have a genotype that is well matched to the changing environment.

**Figure 4.**
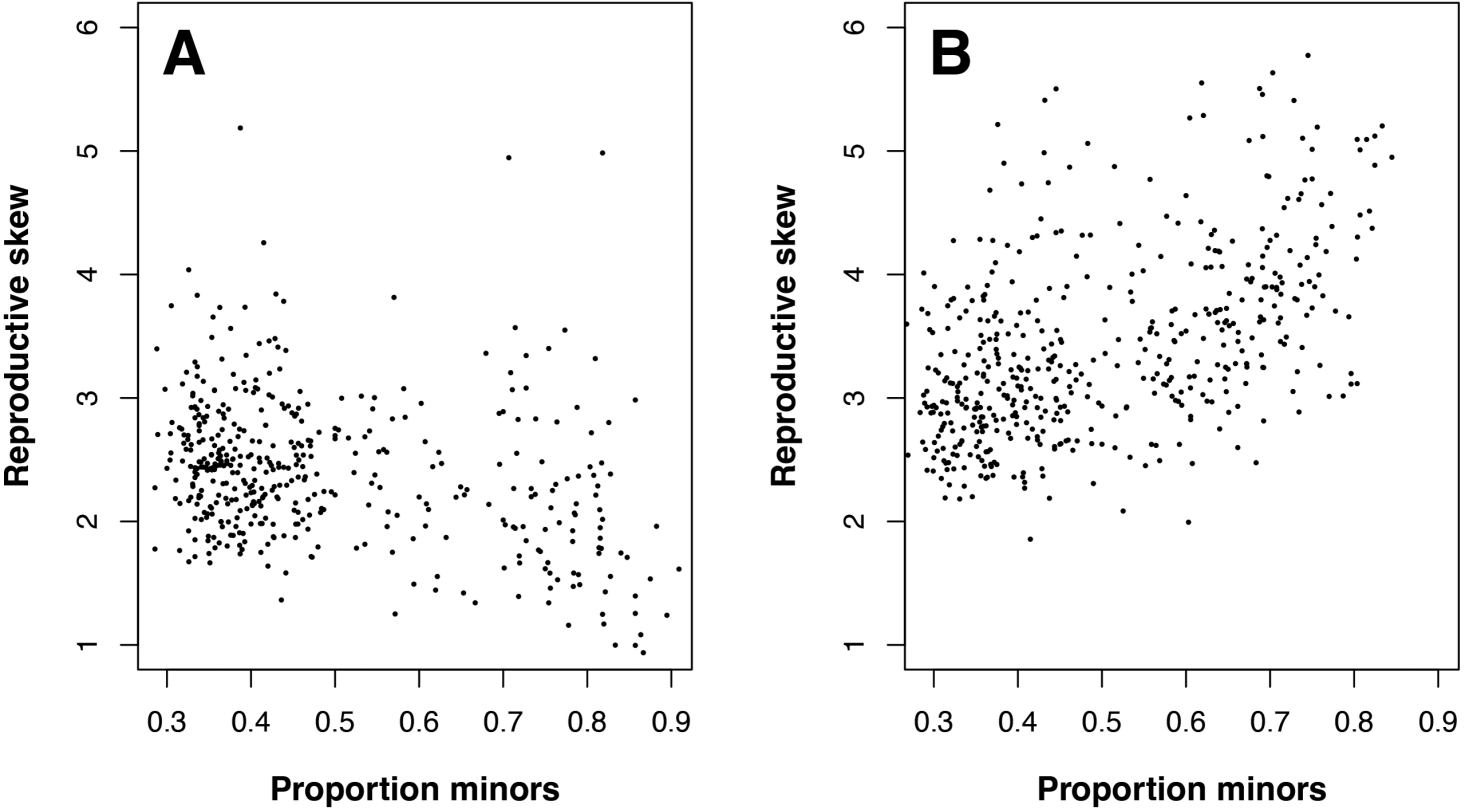
Reproductive skew in males plotted against the proportion of minor males in the population for each time step of a simulation running for 500 time steps. (A) Alternative tactics are simultaneous, and male reproductive skew decreases slightly with the proportion of minor males. (B) Alternative tactics are fixed; male reproductive skew increases with the proportion of males following the minor strategy. Other parameter values: *K* = 500, *ART_success* = 0.5, *max_offspring* = 6, *group_size* = 6, *threshold* = 0.25, and *β* = 2.

To further explore this, we calculated the amount of reproductive skew in males for each time step for simulations with simultaneous and fixed male ARTs. Reproductive skew was calculated as the degree of skewness in the distribution of offspring per male using the moments package in R (Komsta & Novomestky, 2022). These data are shown in Figure 4. While the amount of reproductive skew is weakly negatively correlated with the proportion of minor males in the simultaneous case, when alternative tactics are fixed there is a clear positive relationship between reproductive skew and the proportion of males pursuing the alternative tactic.

## Discussion

ARTs are found across the animal kingdom, so if they have an impact on adaptation and evolutionary rescue, it is important that we understand this. On the basis of this model, we find that whether the ART is simultaneous or fixed is a crucial determinant of the population-wide evolutionary impact of the ART. When ARTs are simultaneous, the capacity of the population to adapt is reduced and evolutionary rescue is less likely when the environment is changing. When ARTs are fixed, however, and males pursuing the alternative tactic do not engage in contests with other males, the adaptive capacity of the population is enhanced and evolutionary rescue becomes more likely.

The reduced adaptive capacity of populations when the ART is simultaneous is intuitively understandable. Poorly adapted males, with a phenotypic optimum that is far from the existing value, will be in poor condition and unlikely to win contests with better-adapted males who are in better condition. By adopting the ART, these males will acquire matings that would otherwise be unavailable to them and will father offspring with maladapted alleles who would otherwise most likely be fathered by better-adapted males, inhibiting the process of adaptation at the population scale. These negative effects will likely be greatest in systems where females are unable to exert much choice over mating rates with males adopting the ART or where there is substantial conflict over matings. For example, male guppies (*Poecilia reticulata*) perform courtship displays in order to solicit matings with females. If, however, females are unreceptive, then males may adopt a coercive mating strategy in which females have little choice over and these coercive copulations have been shown to transfer a considerable amount of sperm (Pilastro & Bisazza, 1999). Although not modeled here, conflict between the sexes over matings, and more broadly sexual conflict, has been shown to have the potential to negate the positive population-level effects of sexual selection (Fricke & Arnqvist, 2007; Holland, 2002; Rundle et al., 2006), and this example adds to that potential. If the coercive ART imposes a cost to females via traits that increase male fitness at the expense of females, then again we would anticipate increased population-level negative effects when males adopt the ART, in a similar way to when harmful male traits are themselves condition dependent (Flintham et al., 2023).

The enhanced adaptive capacity of populations when the ART is fixed, on the other hand, does not seem intuitively obvious. As discussed in the results, this effect occurs because once the males following the ART are removed from competition, the average condition (and therefore degree of adaptation) of males undergoing competition will be higher, leading to greater reproductive skew toward the males in the best condition and consequently a greater representation of alleles conferring better adaptation in the next generation. This effect is robust and persists even when males pursuing ARTs have enhanced probabilities of acquiring matings and fathering offspring (see Supplementary Material).

One approach to thinking about this result is in the context of soft selection versus hard selection (Bell et al., 2021), whereby hard selection implies that an individual’s fitness is largely determined by their phenotypic fit to environmental factors such as temperature or pH, whereas soft selection fitness is determined by an individual’s social environment, meaning that it is their phenotype relative to their conspecifics that determines fitness. In general, males are thought to experience softer selection than females (Li & Holman, 2018), and this is the case in this model. One consequence of this soft selection on males is that a male with a relatively poor genotype in terms of phenotypic fit to the environment can still have high fitness if he is, by chance, in a group of competitors who all have even lower quality genotypes. Introducing the fixed ART hardens selection on males because the least well-adapted individuals are removed from competition, reducing the probability of a low-quality male achieving high fitness because of the chance composition of the group males he is competing with.

This surprising result for fixed ARTs is, of course, a theoretical outcome from a simulation model, and it is necessary to ask whether we should expect this mechanism to operate in real animal populations. There are no empirical studies that we are aware of that would provide a direct hypothesis test, but we can at least examine the assumptions of the model and consider whether there is any further evidence that might help us to address this. The strength of this effect relies on two assumptions in the model: First that the ART is fixed, and second that females will continue to mate with males that are not following the ART even when such males are rare. The first assumption that following the ART is fixed and that males pursuing this tactic do not engage in contests is certainly valid for many systems where ARTs are used—minor dung beetles do not invest in the horns that are necessary for contests with majors (Simmons et al., 2007), for example, and scrambler morphs of the mites *Sancassiana berlesei* and *Rhizoglyphus robini* suffer high mortality from fights (Radwan, 1993; Radwan & Klimas, 2001) and therefore avoid fights with fighter morphs. The second assumption, that when males that are not following the ART are rare, all the females in the population will continue to mate with these males in the same way as when they are common, is weaker. Considering a female-choice system as an example, when such males are very rare some females are likely to mate with low-quality males if they are unable to easily find appropriate high-quality males. Even when females can locate suitable males the strength of female choice in a stressed system could be reduced by both a lack of prior experience (Bailey & Zuk, 2008; Macario et al., 2017) and the female’s condition (Hunt et al., 2005). Similar arguments can be made about systems where the males engage in contests to monopolize females. In the extreme then, and in populations that are especially stressed by the changing environment the strength of the effect we have found is likely to be reduced, but it will not be eliminated altogether. In species where males compete to guard rare resources that are important for female reproduction, such as oviposition sites, then even when only a few high-quality males are present, these will still enjoy a high mating success because of the lack of rivals.

Does the phylogenetic distribution of ARTs tell us anything about whether they might enhance or reduce the persistence of a species? If the presence of males following ARTs reduces species persistence, we might expect to see a “twiggy” distribution of ARTs, with species expressing ARTs being found individually or in small clades at the tips of phylogenies, but with no large taxa expressing them, as has been suggested to be the case for asexual reproduction (Maynard, 1978; Schwander & Crespi, 2009). An analysis of the phylogenetic distribution of ARTs across the animal kingdom is not possible with current knowledge, but we can note that within the Coleoptera, there are a number of large clades with males that seem mostly to pursue ARTs. McCullough et al. (2015) examined the static allometry of the horns carried by males of 31 species from the Dynastinae (Hercules beetles) and found discontinuous relationships suggestive of ART expression in 30 of them. Within the Lucanidae (stag beetles), Matsumoto and Knell (2017) found evidence for complex polymorphisms, including species with three and even four male morphs, in all six species of *Odontolabis* examined. Within the Scarabaeinae (true dung beetles), the picture is somewhat more complex because, in the genus *Onthophagus* at least, male weaponry is known to be evolutionarily labile such that horns are rapidly gained and lost over evolutionary time (Emlen et al., 2005). Nonetheless, when the horned dung beetles are examined, the great majority of species seem to exhibit male polymorphism that is most probably associated with males following either a “guard” or a “sneak” strategy (Parrett et al., in prep.; Simmons et al., 2007). The existence of clades of animals where most or all males seem to pursue ARTs suggests that in the beetles at least the use of these tactics is not a significant contributor to the probability of extinction and is possibly enhancing species persistence. The behavior of the minor males has only been studied in detail in a relatively small number of species from the Scarabaeinae, but it is notable here that these minors all seem to follow something approximating to a fixed strategy—in *O. acuminatus* and *O. taurus*, e.g., minor males will enter burrows and remain there with females, but if challenged by a major male are unable to compete and resort to “sneak” behavior to acquire matings (Emlen, 1997; Moczek & Emlen, 2000). Future work might focus on ART distributions in fish: Both fixed and simultaneous ARTs are found within the ray-finned fish (Taborsky, 1998, 2008) and have been shown to have multiple independent origins (Mank & Avise, 2006). Addressing the distribution of each type of ART across this group may provide a direct test of the possibility that ART type influences extinction risk.

As a final point, we should point out that this model is only appropriate for considering the impact of some types of ART on the adaptive potential of a population. ARTs are diverse, and we have not considered, e.g., sequential ARTs where the reproductive tactic changes through the life of an animal. These are common in fish and mammals (Oliveira et al., 2008) and might have different effects on adaptive potential. ARTs that are based on genetic polymorphisms are very different from the polyphenic type modeled here, and whether these might alter adaptive potential is currently an open question.

## Supplementary Material

qrae010_suppl_Supplementary_Material

## Data Availability

Model code, analysis code, and data from simulations are all available from Dryad at doi:10.5061/dryad.9zw3r22p1.
