# Supplementary material for: Alternative reproductive tactics and evolutionary rescue
Source: Evol Lett. 2024 Mar 16;8(4):539–49. doi: 10.1093/evlett/qrae010 (PMC11291626; doi:10.1093/evlett/qrae010)
Supplement: qrae010_suppl_Supplementary_Material [file qrae010_suppl_supplementary_material.pdf]

Alternative reproductive tactics and evolutionary rescue: supplementary material

Authors: Robert J Knell[1] & Jonathan M. Parrett[2]

[1]Author for correspondence.
School of Natural Sciences, University of Hull, Hull, UK, email: r.j.knell@hull.ac.uk

[2]Evolutionary Biology Group, Faculty of Biology, Adam Mickiewicz University, Poznań, Poland

Table of Contents

Critical rates of environmental change and the strength of sexual selection 2
Effects of increasing the success of minor males 4
Reducing the genetic component of morph allocation 5
Reducing the cost of expressing a sexual signal or weapon 8

Critical rates of environmental change and the strength of sexual selection

To further explore the way that the presence of males using alternative tactics affects evolutionary rescue under directional environmental change, we calculated the Critical Rate of Environmental Change (Chevin et al., 2010)—the rate of environmental change above which a population cannot adapt and persist—for a variety of parameter combinations. Because of the stochasticity in these models there is not a single rate above which a population will persist, so the critical rate was approximated as the rate of environmental change at which 50% of simulations became extinct. This was estimated by running 100 simulations at a range of values for the rate of change (initially 0.002 to 0.008 in steps of 0.001 and then further values from 0.00325 to 0.00575 in steps of 0.0025 in order to achieve more precise estimates) and fitting a generalised linear model with binomial errors to the data on extinction with rate of change as an explanatory variable. The rate of change at which 50% of populations were predicted to become extinct was then extracted from each model using the *dose.p()* function of the MASS package running in R (Venables and Ripley, 2002).

Figure S1 shows the effect of including fixed or simultaneous alternative tactics and the size of the mating groups for three values of *β*, the parameter that controls the probability of the highest ranked male acquiring a mating in a particular mating group. Both larger group sizes and higher values of *β* will lead to stronger sexual selection in the population, and values for the critical rate are highest when both of these values are high. For simultaneous alternative tactics, which, as we have seen, tend to decrease persistence in changing environments the effect size (the magnitude of the decrease in the critical rate) is largest when sexual selection overall is strong, with the largest decreases in the critical rate of environmental change when *β* = 4 and *group.size* = 10. There is also an interaction between the strength of sexual selection and *threshold*, the parameter that specifies the value of *condition* below which a male develops into a minor male, such that the critical rate of environmental change decreases much more with strong sexual selection when *threshold* = 0.5 than when *threshold* = 0.25.

When the alternative tactics are fixed then the effect of introducing alternative tactics is positive, meaning that the critical rate of environmental change is greater when males in the population pursue alternative tactics. When the threshold for pursuing alternative tactics (*threshold*) is set at a *condition* of 0.25 then introducing the fixed ART leads to a consistent increase in the critical rate of roughly 0.001 no matter what the strength of sexual selection, but when *threshold* is 0.5 the extra increase in the critical rate arising from the alternative tactic is reduced when sexual selection is strongest, in other words when *group_size* is 8 or 10 and *β* is 4. The reason for this is not clear but we suggest that ultimately adaptation in the population will be limited by other factors such as the population size and the average magnitude of mutations: if the environment is changing very rapidly then there will simply not be variation being added to the *environmental genotype* to allow adaptation no matter what the mating system.

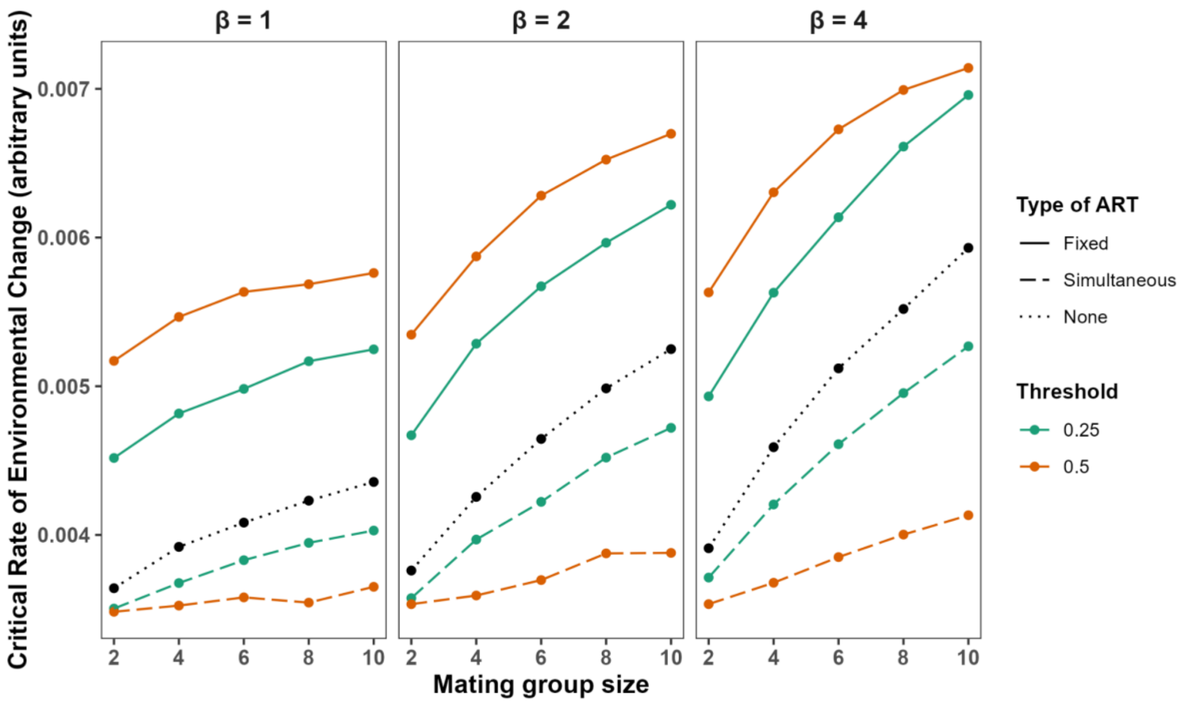

Figure S1: variation in the critical rates of environmental change with increasing strength of sexual selection for populations without ARTs and for populations with males expressing fixed or simultaneous ARTs. The two parameters that between them determine the strength of sexual selection in a simulated population are *group_size*, which determines the size of the groups of males where competition takes place, and *β*, which determines the probability of the highest ranked male in each group 'winning'. Threshold indicates the condition threshold below which a male will follow alternative mating tactics. Higher values for the critical rate of environmental change of a population indicate that the population can persist when environmental change is more rapid. All simulations were run for 500 time steps and other parameter values were *K_cap* = 500, *max_offspring* = 6, *and ART_success* = 0.5.

Effects of increasing the success of minor males

Figure S2 shows the effect of increasing the success of minor males in acquiring mates via the ART. As can be seen, increasing the number of matings that these males achieve does reduce the critical rate of environmental change, whether the ART is fixed or simultaneous. Notably, however, even with substantial increases in the mating success of males pursuing the ART the critical rate remains higher than it would be in the absence of such males when the ART is fixed. Indicating that this effect of fixed ARTs is robust even when the males pursuing the alternative tactic are able to gain matings successfully.

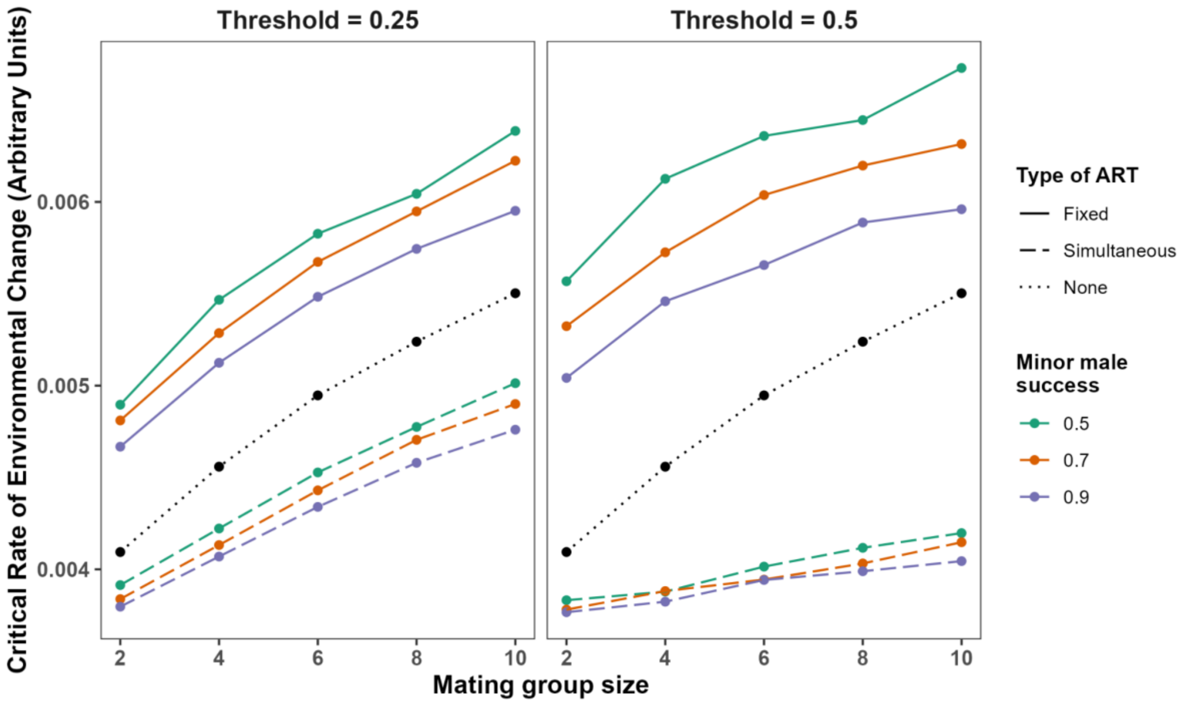

Figure S2: the effect of changing the probability of success for males pursuing alternative tactics on the critical rate of environmental change. "Minor male success" is the parameter *ART_success* from the simulation which determines the probability of a male pursuing the alternative tactic achieving a mating. Mating group size is the parameter *group_size*, which determines the size of the groups of males where competition takes place. Threshold indicates the condition threshold below which a male will follow alternative mating tactics. Higher values for the critical rate of environmental change of a population indicate that the population can persist when environmental change is more rapid. All simulations were run for 500 time steps and other parameter values were *K_cap* = 500, *max_offspring* = 6, *and* β = 2.

Reducing the genetic component of morph allocation

As discussed in the introduction to the main paper, there is some uncertainty over the degree by which morph allocation is heritable in many species which exhibit ARTs. In the model presented in the main manuscript, morph allocation is determined by condition, where condition is calculated as:

$$condition = resource - |environmental\ genotype - environment|$$

where *resource* represents a randomly determined environmental component. Males with a value for *condition* which is below a threshold value will become minor males, and those with a value equal to or greater than the threshold will become majors. While this already includes a strong non-genetic competent in morph allocation, we here explore the effects of reducing the genetic component even more. This was done by multiplying the difference between the *environmental genotype* and the variable *environment* by either 0.5 or 0.1 before morph allocation. After morph allocation *condition* was recalculated using the original formula for purposes of determining display trait expression in majors, mortality and reproduction. This has the effect of uncoupling morph allocation from the genetic fit of an individual to the environment while retaining the negative effects of a poor fit in other aspects of the individual's life history.

Figure S3 shows the effect of reducing the genetic component of morph allocation when the ART is simultaneous. As the genetic contribution to morph allocation is reduced so the effect of the presence of the ART is also reduced and when the genetic contribution is very small then there is no visible effect—the presence of the ART no longer counteracts the effect of stronger sexual selection in promoting population persistence. When the ART is fixed, however, the effect of the ART on population persistence is reduced but not eliminated even when the genetic contribution to morph allocation is very small (figure S4). This latter finding is consistent with other results indicating that this effect of fixed ARTs seems to be strong and robust, so even when there is only a very small correlation between an individual's degree of adaptation to the environment and morph allocation, the removal of the worst adapted individuals from competition for mates continues to have an effect.

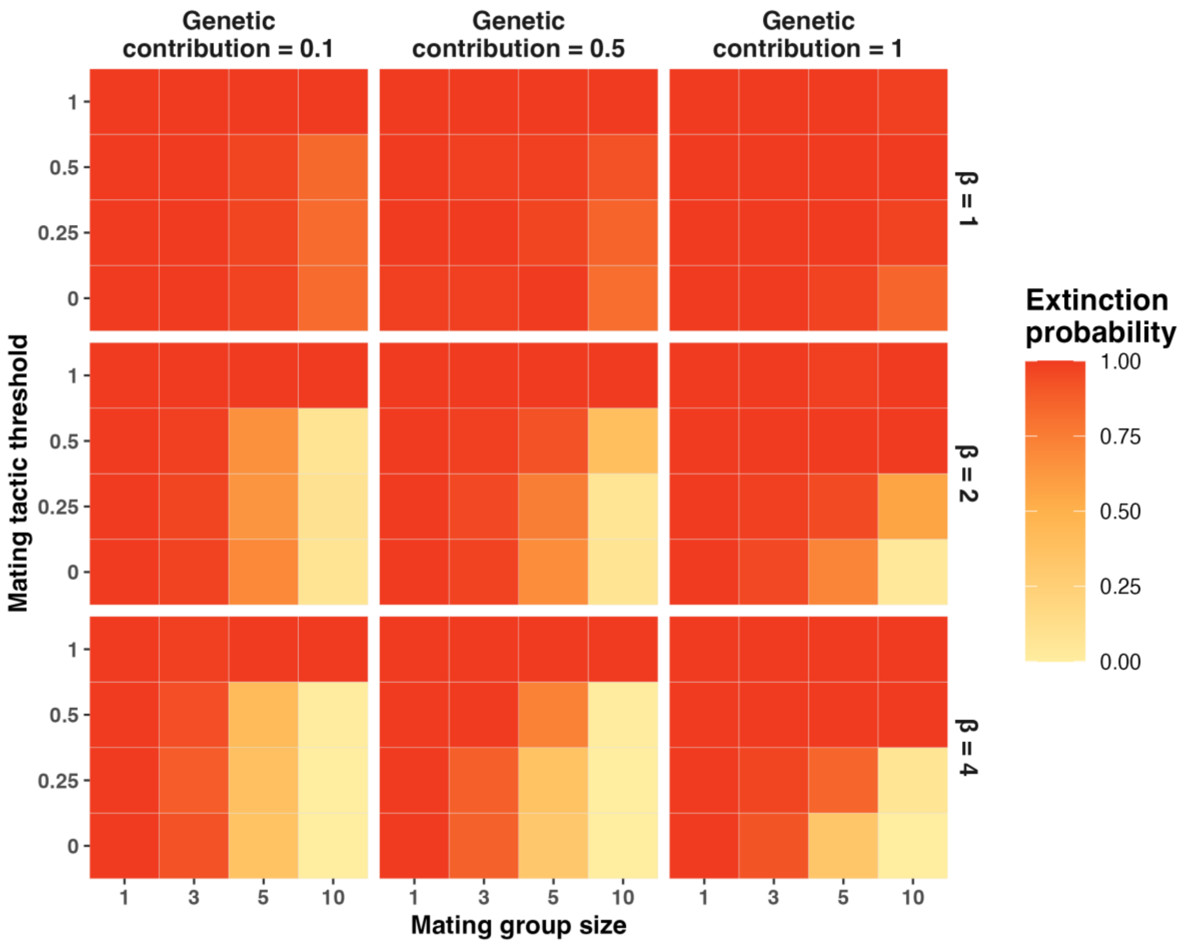

Figure S3: the effect of reducing the contribution of genetic factors to morph allocation. The heatmap shows the probability of extinction under directional environmental change when alternative tactics are simultaneous, calculated from 100 simulations running for 500 time steps for each combination of parameter values. The rate of directional change was set to 0.005 for all simulations. “Genetic contribution” refers to the value by which the absolute difference between the genotype and the environment variable was multiplied by before calculating morph allocation. Other parameter values: max_offspring = 6, ART_success = 0.5, carrying capacity = 500.

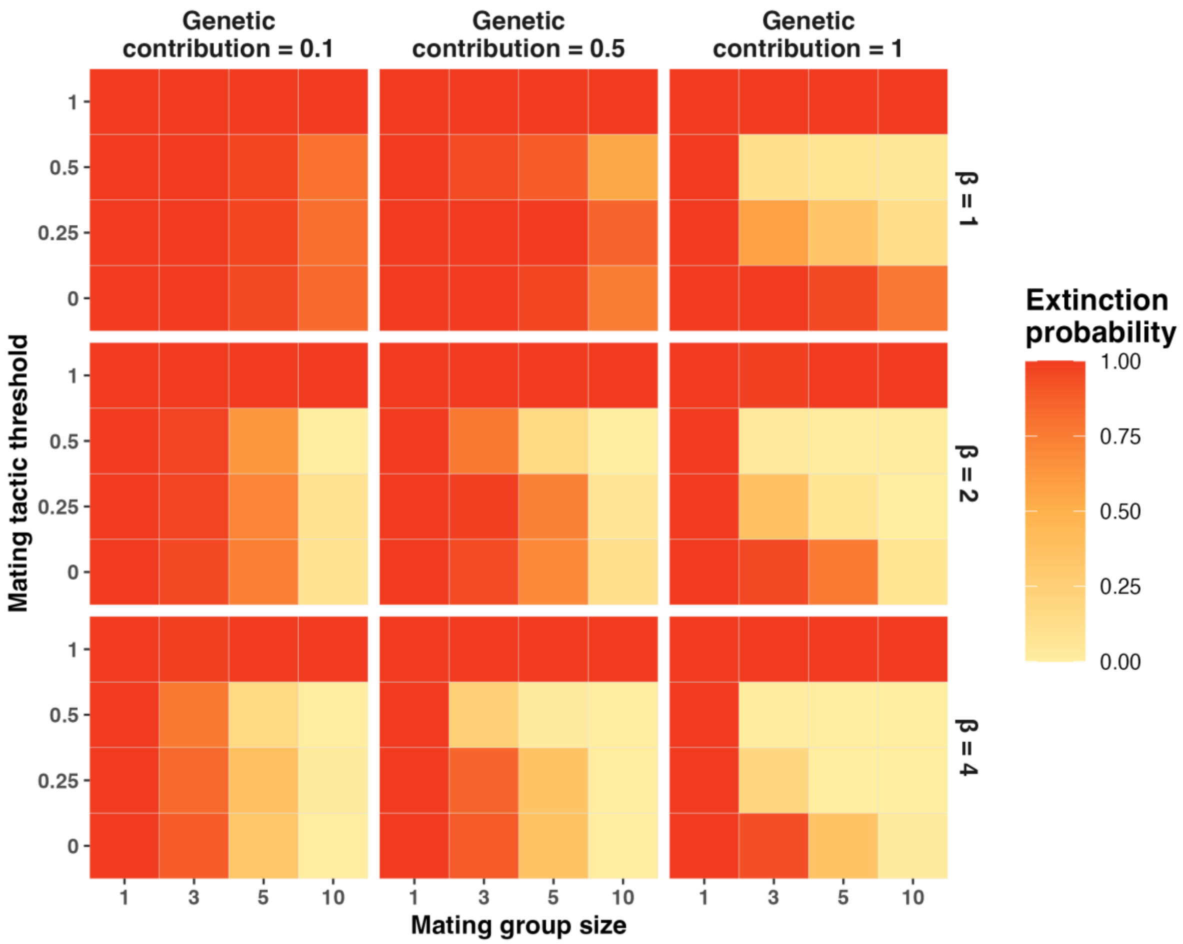

Figure S4: the effect of reducing the contribution of genetic factors to morph allocation. The heatmap shows the probability of extinction under directional environmental change when alternative tactics are fixed, calculated from 100 simulations running for 500 time steps for each combination of parameter values. The rate of directional change was set to 0.005 for all simulations. "Genetic contribution" refers to the value by which the absolute difference between the genotype and the environment variable was multiplied by before calculating morph allocation. Other parameter values: max_offspring = 6, ART_success = 0.5, carrying capacity = 500.

Reducing the cost of expressing a sexual signal or weapon

Figure S5 shows a set of heatmaps comparing model outcomes when the cost to males of display or competition is set to zero with the default value (two) used in the main manuscript. As can be seen, there are some quantitative changes to the output with population persistence being somewhat more likely when there is no cost to pursuing the “major” tactic, the qualitative results form the model are unchanged.

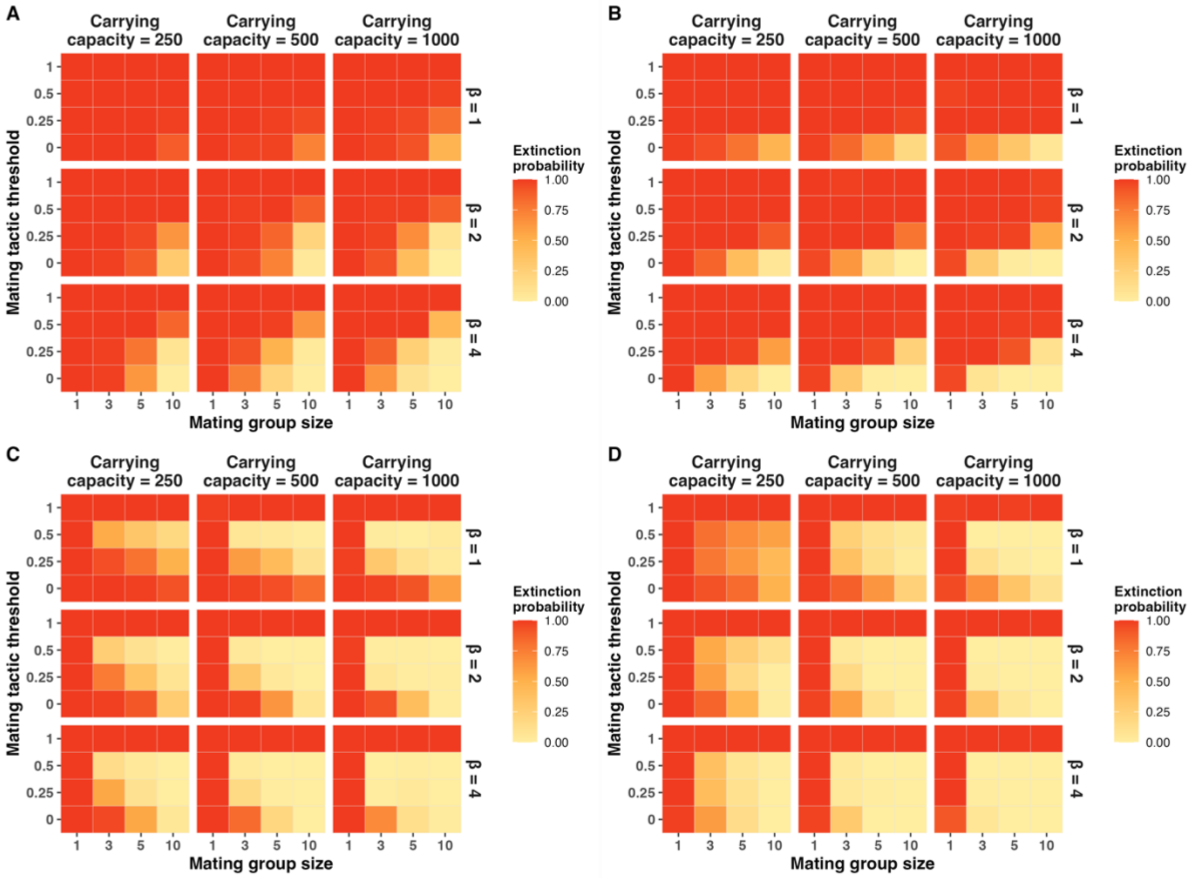

Figure S5: probabilities of extinction when the cost of pursuing the “major” tactic is set to zero or set to two (as in the ms). A: cost = zero, ART is simultaneous. B: cost = two, ART is simultaneous. C: cost = zero, ART is fixed. D: cost = two, ART is fixed. Other parameter values: max_offspring = 6, ART_success = 0.5.

Chevin L-M, Lande R, Mace GM. 2010. Adaptation, Plasticity, and Extinction in a Changing Environment: Towards a Predictive Theory. *PLoS Biol* **8**:e1000357.
Venables WN, Ripley BD. 2002. Modern Applied Statistics with S.
